# Complications and clinical outcome of hepatic artery embolisation in patients with hereditary haemorrhagic telangiectasia

**DOI:** 10.1007/s00330-012-2694-9

**Published:** 2012-10-31

**Authors:** Ajay Chavan, Lars Luthe, Michael Gebel, Hannelore Barg-Hock, Hans Seifert, Rudolph Raab, Timm Kirchhoff, B. Schmuck

**Affiliations:** 1 Department of Diagnostic and Interventional Radiology, Klinikum Oldenburg, Rahel Straus Strasse 10, 26133 Oldenburg, Germany; 2 Department of Gastroenterology and Hepatology, Hannover Medical School, Hannover, Germany; 3 Department of Abdominal and Transplantation Surgery, Hannover Medical School, Hannover, Germany; 4 Department of Gastroenterology, Hepatology and Diabetology, Klinikum Oldenburg, Oldenburg, Germany; 5 Department of General and Visceral Surgery, Klinikum Oldenburg, Oldenburg, Germany; 6 Department of Radiology, Hannover Medical School, Hannover, Germany

**Keywords:** Embolisation, Therapeutic, Arterio-venous malformations, Cardiac failure, Biliary disease, Liver transplantation

## Abstract

**Background:**

Hepatic artery embolisation (HAE) in patients with hereditary haemorrhagic telangiectasia (HHT) is controversial because of the associated complications and unproven long-term benefit. We present our results in 20 such patients over a time span of 17 years.

**Methods:**

Staged HAE was performed using polyvinyl alcohol (PVA) particles and coils. Complications, clinical symptoms and cardiac output were assessed before and after therapy as well as at the end of follow-up (median 92 months, range 26–208 months).

**Results:**

Two patients died within 30 days following HAE (10 %). Four further deaths resulted from causes unrelated to HAE. Ischaemic cholangitis, cholecystitis and focal hepatic necrosis with biliary sepsis necessitated re-intervention in four patients. In all but one patient, clinical symptoms resolved with mean cardiac output falling from 11.84 ± 3.22 l/min pre-treatment to 8.13 ± 2.67 l/min at the end of follow-up (*P* < 0.001). One patient required liver transplantation for de novo symptoms of portal hypertension 4 years after primary symptoms had been cured by HAE.

**Conclusion:**

The 30-day mortality of HAE in patients with HHT is 10 %. The rate of complications requiring re-intervention is 20 %. Clinical response at long-term follow-up is satisfactory.

***Key Points*:**

• *Hepatic artery embolisation (HAE) in hereditary haemorrhagic telangiectasia (HHT) provides long-term benefit.*

• *Mortalities of HAE and liver transplantation in HHT patients are comparable.*

• *In HHT, complications of HAE are lower than those of liver transplantation.*

• *Complications of HAE can be further reduced by refinement of technique.*

• *Complications include ischaemic cholangitis, hepatic necrosis, biliary sepsis and death.*

## Introduction

The prevalence of hepatic involvement in hereditary haemorrhagic telangiectasia (HHT) has been estimated to be between 41 % and 78 % with a clear female predominance. Most patients are asymptomatic [1, 2]. Symptomatic hepatic involvement that cannot be managed medically necessitates intervention [3, 4]. Currently, liver transplantation and hepatic artery embolisation (HAE) are the two main treatment options. Consensus recommendations published in 2006 [3] as well as the international guidelines published in 2009 [5] favour liver transplantation over HAE on account of the procedure-related complications of HAE, which may even lead to liver transplantation [6] or death [7]. In an attempt to define the role of HAE in the treatment algorithm of such patients, we present the complications, clinical outcome and the quality-of-life assessment in 20 consecutive patients who were treated with HAE over a time span of 17 years.

## Materials and methods

Between May 1991 and April 2007, 20 of the 38 patients presenting at the Hannover Medical School or at the Klinikum Oldenburg (teaching hospital affiliated to the Göttingen University Medical School) having HHT with symptomatic hepatic involvement were treated with staged HAE after reaching an interdisciplinary consensus between the radiologists, gastroenterologists and transplant surgeons. Pertinent follow-up records until January 2009 were evaluated. The diagnosis of HHT was made using the Curaçao criteria [8]. At least three Curaçao criteria were present in all patients. Informed consent was obtained from all patients. The median follow-up period was 92 months (range 26–208 months). Short and mid-term results in 15 of the 20 patients have been reported previously [9, 10].

The inclusion criteria were therapy-resistant right-upper-quadrant capsular pain or abdominal angina, portal hypertension with ascites, pedal oedema and/or oesophageal varices and a raised cardiac output exceeding 7 l/min associated with clinical features of cardiac failure/insufficiency including early fatigue, limitation of physical activity, dyspnoea, orthopnoea or pedal oedema. Asymptomatic patients or those who could successfully be treated conservatively with medical therapy were excluded from the study. All patients fulfilling the inclusion criteria underwent HAE. On account of the severity of their symptoms, all 20 patients requested or demanded therapy; consequently, there were no therapy refusals.

The diagnostic workup included colour Doppler ultrasound of the liver as well as helical CT of the entire abdomen before and after therapy and at the end of follow-up. Routine laboratory parameters as well as those specific to the liver were recorded before and after every intervention as well as at the end of follow-up. Cardiac output was determined using echocardiography prior to therapy and at the end of follow-up. In patients who died, the last cardiac output measured prior to death was used for purposes of statistical evaluation.

Hepatic artery embolisations in stages were carried out in one to five sessions at intervals ranging from 1 to 15 weeks. The intervals between the embolisation sessions were determined by the time required for the patient to recover from the previous embolisation as well as by the time required for the hepatic parameters to return to pre-embolisation levels.

Branches of the right or left hepatic artery were selectively or super-selectively catheterised using 5-F diagnostic catheters with or without 3-F co-axial catheters in addition. The peripheral vascular bed was first embolised with a mixture of polyvinyl alcohol (PVA) particles of sizes ranging between 250 and 1,180 μm followed by embolisation of the branch vessel with platinum microcoils (Target Therapeutics, Fremont, CA, USA) or steel macrocoils (William Cook Europe A/S, Bjaerverskov, Denmark). With the exception of one patient who underwent five embolisation sessions, only tertiary branches of the hepatic arteries (and beyond) were embolised, leaving the central vessels open. The vessels in the right and the left lobe were embolised in separate sessions. During the procedure, the patient received an infusion containing an analgesic, an anti-emetic and a steroid. An embolisation session was concluded either when the patient developed right upper quadrant or epigastric pain or when the angiogram showed marked reduction or disappearance of the peripheral telangiectasia in the portion of the liver that was embolised. Prophylactic antibiotic coverage was provided for at least 5 days following the procedure and longer if clinically indicated. Analgesics and anti-emetics were administered as and when necessary during and after every embolisation. Further procedural details have been described in a previous publication [9].

The quality of life before HAE and at the end of follow-up was assessed retrospectively in surviving patients with the help of a modified SF 36 questionnaire in order to document subjective patient response. Data assessed by the questionnaire included information relating to fatigability, pain and general constitution, which were classified by the patients as poor, unsatisfactory, satisfactory, good or excellent.

### Statistical analysis

Continuous variables are expressed as means ± SD. Comparison of cardiac output before treatment and at the end of the observation period was performed applying Student’s paired *t*-test and Fisher’s exact test (SPSS for Windows 10.0; SPSS, Chicago, IL); *P* values < 0.05 were considered statistically significant.

## Results

### Patient demographics and pre-procedural symptomatology (Table 1)

Of the 20 patients, 4 were men and 16 were women with ages ranging between 29 and 72 years (mean 53.7 ± 9.53 years).

**Table 1:** Patient demographics and clinical features before hepatic artery embolisation (HAE) as well as at the end of follow-up

Pat no.	Patient age (years)	Sex	Capsular pain		Abdominal angina		Portal hypertension		Hepatic encephalopathy		Cardiac output (l/min)		Remarks
			Before	After	Before	After	Before	After	Before	After	Before	After	
1	53	F									11.8	9.0	
2	49	F	++	−							11.4	8.0	
3	57	F									8.2	4.5	
4	72	M									13.5	12.4	Died 3 months after 2 sessions of HAE of complications of severe Parkinsonism and fracture neck femur
5	56	F	++	−							7.4	4.8	
6	57	F							+	+	7.6	8.1	Died within 30 days after third HAE
7	29	F			+++	−					14.0	7.5	Two uncomplicated pregnancies after treatment
8	54	F					+	−			15.0	9.7	
9	41	F					+	−			17.3	5.8	Died 7 months after completion of HAE of post-operative sepsis following laparotomy
10	59	M	++	−							12.0	9.2	
11	41	F			+++	−					12.9	6.3	
12	47	F									12.5	8.0	
13	61	F									10.1	9.0	Died 36 months after completion of HAE following hip surgery
14	56	F					+	+	+	+	10.0	11.0	Died within 30 days after first HAE
15	63	F	++	±			+	−			7.5	5.5	
16	52	F									10.7	8.0	
17	50	M									19.5	15.0	
18	67	M							+	+	14.0	8.8	Died 5 months after 3 sessions of HAE of severe encephalopathy and nasal bleeding
19	48	F	++	±							9.1	4.2	
20	61	F									12.3	7.7	

*Before* pre-therapy; *after* post-therapy at end of follow-up; + present; − absent; ± occasionally present

The cardiac output was raised in all patients and ranged between 7.4 l/min and 19.5 l/min (mean 11.84 ± 3.22 l/min).

Cardiac failure/insufficiency with symptoms such as early fatigue, limitation of physical activity, dyspnoea, orthopnoea and pedal oedema was present in 16 patients.

Of the seven patients with therapy resistant abdominal pain, two had abdominal angina presenting post-prandially and five had capsular pain caused by hepatomegaly and vascular congestion of the liver.

Ascites, oesophageal varices and pedal oedema as signs of portal hypertension were present in four patients.

Three patients suffered from hepatic encephalopathy.

A combination of two of the manifestations stated above was present in six patients and of three manifestations in three patients.

### Procedural complications (Table 2)

Post-interventional abdominal pain in the epigastric region and/or in the right upper quadrant was experienced by all but one patient. Under a combination of analgesics consisting of metamizol, tramadol and opiates, the pain resolved after 2 weeks in 14 patients and after 6 weeks in one patient.

**Table 2:** Complications of HAE in 20 patients along with the interventions performed for treating them

Pat. no.	Patient age		Complication					Surgical intervention
			Ischaemic cholecystitis	Ischaemic cholangitis	Hepatic necrosis, biliary abscess	Biliary sepsis	Acute pancreatitis	
5	56	F	+		+			- Debridement of hepatic necrosis
								- Cholecystectomy
2	47	F	+	+			+	- Cholecystectomy
								- (Rest managed conservatively)
13	61	F	+		+			- Debridement of hepatic necrosis
								- Cholecystectomy
19	48	F	+	+	+	+		- Debridement of hepatic necrosis
								- Cholecystectomy
								- Partial hepatic resection

+, present

**Fig. 1:**
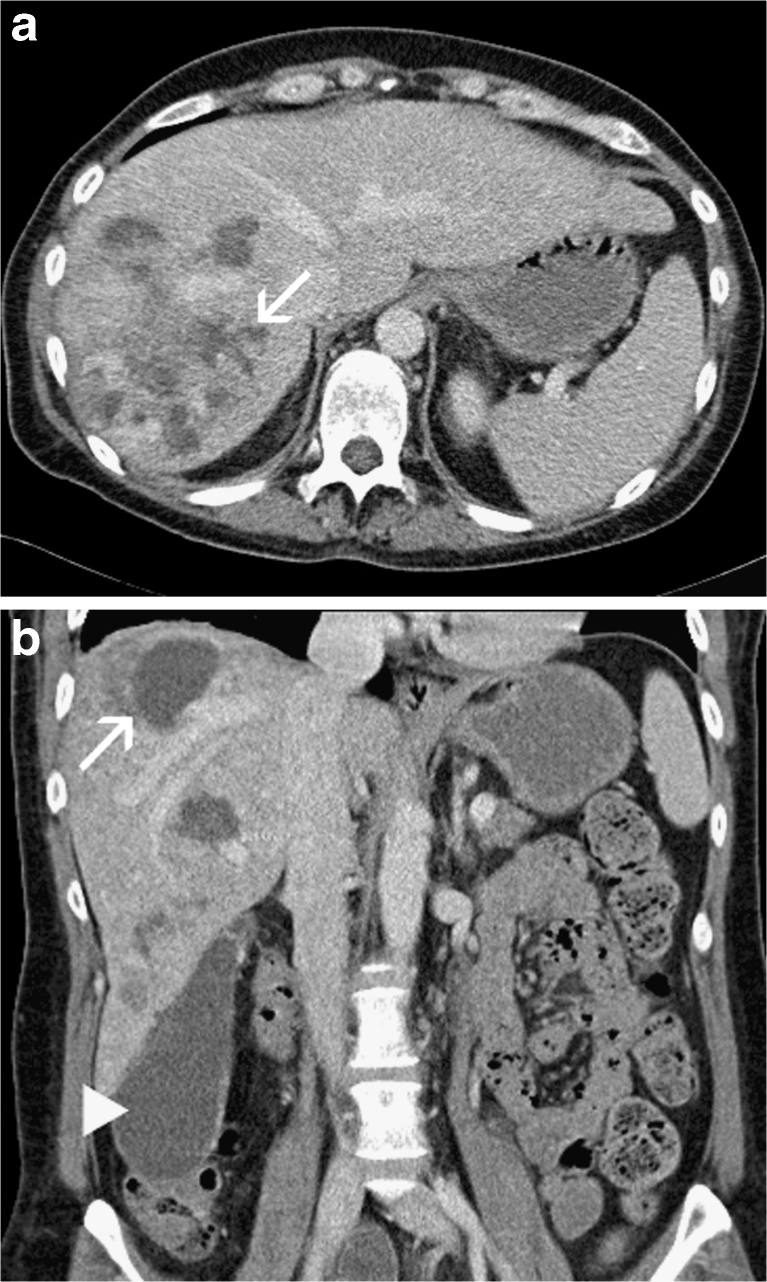
CT in the portal venous phase, axial section (**a**) and coronary reconstruction (**b**) showing multiple biliary abscesses (*arrows*) and hydrops of the gall bladder (*arrowhead*) 22 weeks following hepatic artery embolisation (HAE)

Ischaemic cholecystitis and cholangitis were observed in four patients; in addition, one of these patients developed an acute pancreatitis, which, however, could be managed conservatively. Focal areas of hepatic necrosis were noted in three of these four patients with multiple biliary abscesses and biliary sepsis in one of them (Fig. 1). All four underwent cholecystectomy 12, 14, 18 and 28 weeks after the last embolisation, with or without concomitant debridement of areas of hepatic necrosis or partial hepatic resection. The surgery was uneventful in three patients. On account of post-operative biliomas, a cutaneous biliary fistula and recurrent bouts of biliary sepsis, the fourth patient was listed for liver transplantation. However, on account of a Model of End-stage Liver Disease (MELD) score of 10 at this stage, transplantation was not performed. Following multiple percutaneous drainages and antibiotic therapy, the patient underwent complete clinical recovery and the MELD score improved to 7.5; consequently, she was taken off the transplantation list 14 months following HAE.

### Thirty-day mortality (Table 1)

Two patients died within 30 days after HAE, giving a 30-day mortality of 10 %. Both patients presented pre-terminally and were in any case deemed unsuitable for liver transplantation on account of severe co-morbidities; the HAE was carried out as “ultima ratio” for lack of other therapy options.

The first patient presented with advanced biliary disease with cirrhosis accompanied by severe hepatic encephalopathy and portal hypertension; in addition, she suffered from chronic tachy-arrhythmias, pleural effusions with marked dyspnoea and pulmonary hypertension with a pulmonary arterial pressure of 50 mmHg [cardiac insufficiency, New York Heart Association (NYHA) IV]. She died of multiorgan failure 23 days after the first embolisation.

The second patient had limited pulmonary reserve due to a previous left-sided pneumonectomy; in addition, pulmonary arteriovenous shunts and pulmonary hypertension were present. She was admitted with cardiac insufficiency (NYHA IV) and biliary disease with cirrhosis accompanied by encephalopathy. Thirty days following the third embolisation, she died of gastrointestinal bleeding and multiorgan failure.

### Further deaths at follow-up (Table 1)

During follow-up, 4 further patients died of causes unrelated to the HAE.

The first one responded well clinically to the HAE with a fall in cardiac output from 17.3 l/min to 5.8 l/min, disappearance of massive ascites and regression of oesophageal varices from grade III to grade I. Seven months following HAE, she was readmitted for critical variceal bleeding. She died of post-operative sepsis following an unsuccessful attempt to create a porto-systemic shunt.

In the second patient, the cardiac output fell from 14.0 l/min to 8.8 l/min and the symptoms of cardiac insufficiency improved after HAE. A bout of grade III encephalopathy accompanied by severe epistaxis led to multi-organ failure and death 5 months after the HAE. At the final admission, further invasive treatment in any form was refused by the patient and her relatives.

The third patient died before completion of therapy. The patient succumbed to complications of severe Parkinsonism and of a fractured neck femur, which occurred before the planned third HAE session.

The fourth patient died of post-operative complications following hip surgery 36 months after successful HAE.

### Clinical outcome (Table 1)

There was a transient increase of serum aminotransferases following embolisation, which returned to pre-embolisation levels at or shortly following discharge. Other than this, there was no significant long-term impairment of liver synthetic function.

Two patients had intractable abdominal angina (caused by a steal phenomeon) with severe resultant weight loss. Following treatment, the abdominal angina resolved and the patients gained 6 and 10 kg body weight respectively in the year following the conclusion of therapy. One of them had two uneventful pregnancies at the age of 34 and 38 years, 31 and 79 months after HAE respectively.

Of the five patients with capsular pain as a presenting feature, three are presently free of pain without medication. Two further patients have been successfully weaned off regular analgesics; only occasional intake of oral analgesics is necessary.

The mean cardiac output reduced significantly (*P* < 0.001) from 11.84 ± 3.22 l/min pre-treatment to 8.13 ± 2.67 l/min at the end of follow-up. Except in the two patients who died within 30 days after the first embolisation, symptoms arising from cardiac failure resolved or improved markedly in all patients.

Of the four patients with portal hypertension, ascites and oesophageal varices disappeared completely in two patients. The third patient responded well to the HAE with a fall in cardiac output, disappearance of massive ascites and regression of oesophageal varices. She however succumbed 7 months later to complications of surgical intervention for critical variceal bleeding. The fourth patient died within 30 days of the procedure.

Worthy to note is that two patients developed de novo signs of portal hypertension 18 months and 4 years after HAE. The first one developed grade I–II oesophageal and fundal varices without ascites. As the patient is asypmtomatic, she is being managed conservatively at present. The second patient developed grade I–II oesophageal varices and therapy-refractory ascites 4 years after HAE. She underwent successful liver transplantation and presently continues to be asymptomatic.

**Fig. 2:**
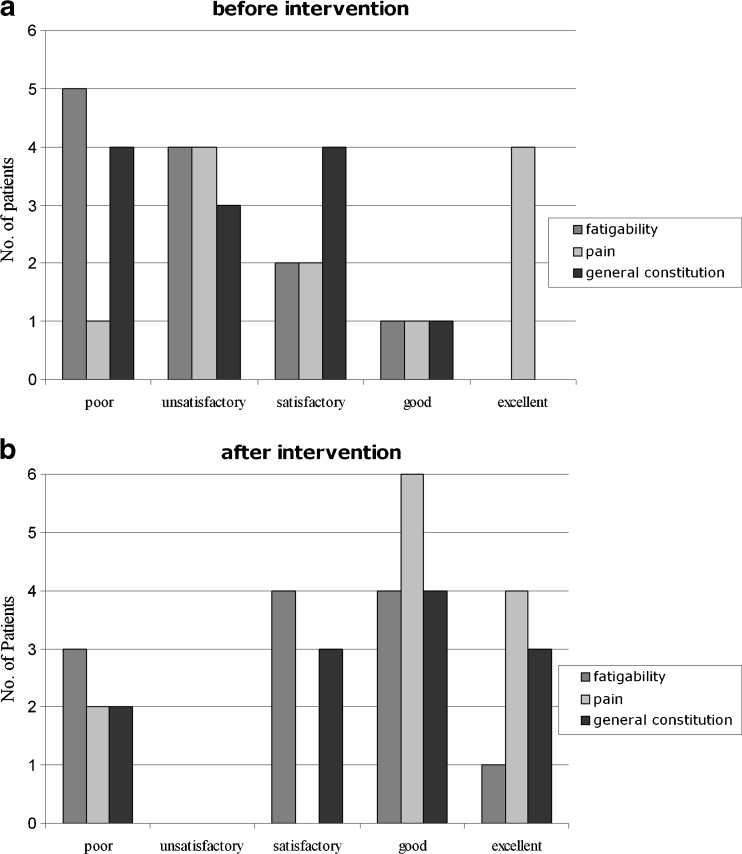
Subjective patient assessment of clinical status with regard to fatigability, pain and general condition before (**a**) and after (**b**) hepatic artery embolisation (HAE)

Analysis of the quality of life questionnaire showed that, prior to therapy, the patients classified their condition on 21 counts as “poor” or “unsatisfactory” with regard to fatigability, pain and general condition. Following therapy, this count fell to seven. In the group consisting of “satisfactory”, “good” or “excellent”, 15 counts were noted pre-therapy; this count improved to 29 post-therapy (Fig. 2).

## Discussion

Therapy-related death occurring within 30 days following HAE was observed in two patients. Both had severe co-morbidities on account of which they were in any case deemed unsuitable for liver transplantation. Amongst the comorbidites in these two patients, pulmonary hypertension, cardiac failure and hepatic encephalopathy resulting from biliary disease and cirrhosis were common to both. Treatment of this set of pre-terminal patients poses a problem to the transplant surgeon and to the interventionist alike. In this respect, the HHT self-help organisations could be of assistance in detecting and treating the patients at an earlier stage.

The overall long-term mortality in our 20 patients amounted to 30 %. This is comparable to the 20 % mortality reported by Lerut and colleagues in 40 patients with liver transplantation [11]; their peri-operative mortality was 17.5 % as compared to the 10 % procedure-related mortality in our patients. Similarly, of the six transplanted patients reported by Azoulay and colleagues, two died [12]. As opposed to this, Dupuis-Girod reported a mortality of 8 % in 13 of their 753 patients (1.7 %) who underwent liver transplantation [13].

It is worthy of mention that two of our four patients with portal hypertension died and that three of the six patients who died had signs of biliary disease with hepatic cirrhosis and encephalopathy. The presence of portal hypertension and cirrhosis with encephalopathy could be poor prognostic factors for patients undergoing HAE, especially if they are further complicated by pulmonary hypertension and cardiac failure.

As is evident from 15 of our 20 patients, uncomplicated post-procedural pain usually resolves within 2 to 6 weeks with conservative treatment with analgesics such as metamizol, tramadol and opiates.

Our procedure-related complication rate of 20 % compares favourably with the 60 % complication rate reported by Lerut and colleagues [11] as well as with the 54 % (7 of 13 patients) experienced by Dupuis-Girod [13]. Six of the documented 35 patients in Lerut’s cohort required additional surgery [11] as was the case in 4 of our 20 patients.

The main complications of HAE are ischaemic cholangitis, ischaemic cholecystitis and focal biliary or hepatic necrosis with or without biliary sepsis [6, 10].

Surgical intervention in the form of cholecystectomy and partial liver resection was necessary in 4 of the 20 patients. Attempts at optimising the HAE procedure should be aimed at reducing these complications. A possible solution would be to embolise smaller portions of the liver at each sitting and to increase the interval between the embolisation sessions. This would avoid injury to large portions of hepatic tissue at a time [10] and give the patient time to recover adequately from the ischaemic insult of the previous procedure. Both of these in turn could prevent the occurrence of widespread biliary sepsis and acute liver failure leading to death [7, 14] or necessitating emergency liver transplantation [6, 15].

Furthermore, during the procedure, it could be worthwhile to place the tip of the embolisation catheter securely distal to the origin of the cystic artery to reduce the incidence of ischaemic cholecystitis; with the large variety of wide lumen micro-catheters available nowadays, this should no longer pose any great difficulty.

The management of HHT patients with hepatic involvement can be complex [1, 2]. As illustrated by Buscarini and colleagues in 154 patients, hepatic involvement is not uncommon in patients with HHT. In these patients, the course of the disease is complicated in about 25 % of the patients who then require treatment. As medical management occasionally supplemented by cardioversion and radiofrequency catheter ablation yield complete (63. 7 %) or partial (21. 8 %) response in the majority of the patients, such management appears to be the most appropriate primary treatment [1, 2]. Further invasive therapy in the form of HAE or liver transplantation should be considered in non-responders to medical management as well as in those with unsatisfactory partial response.

Similar to liver transplantation, a good clinical response with respect to amelioration of abdominal angina, capsular pain and symptoms of cardiac decompensation or portal hypertension can be expected at long-term follow-up after HAE. The procedure-related mortality rates with both the procedures are comparable, whereas complication rates are lower with HAE. Should one be successful in further reducing the complication rate with the methods suggested above, HAE would become an even more attractive therapy option for treating patients with HHT and symptomatic hepatic involvement, at least till such time as the role of vascular endothelial growth factor (VEGF) antagonists in the long-term treatment of these patients becomes better defined [16–18]. This is especially important as patient reluctance to undergo major surgery in the form of liver transplantation often influences the choice of therapy. However, patients with concomitant portal hypertension and cirrhosis with encephalopathy, especially if further complicated by pulmonary hypertension and cardiac failure, could be a high-risk group for HAE. This subset of patients could possibly benefit more from primary liver transplantation, should they be suitable for it.

## Abbreviations

HAE: Hepatic artery embolisation
HHT: Hereditary haemorrhagic telangiectasia
PVA: Poly vinyl alcohol
MELD: Model of End-stage Liver Disease
NYHA: New York Heart Association
VEGF: Vascular endothelial growth factor
